# Neurodivergent patient experience in a tertiary children's hospital-a qualitative analysis

**DOI:** 10.3389/fped.2024.1427433

**Published:** 2024-07-16

**Authors:** Michele Talley, Chelsea Brown, Nancy Wingo, Jennifer Conway, Julian Maha, Michele Kong

**Affiliations:** ^1^ School of Nursing, University of Alabama at Birmingham, Birmingham, AL, United States; ^2^ Children’s of Alabama, Birmingham, AL, United States; ^3^ KultureCity, Birmingham, AL, United States; ^4^ School of Medicine, University of Alabama at Birmingham, Birmingham, AL, United States

**Keywords:** neurodiversity, sensory processing differences, pediatrics, hospital, sensory needs

## Abstract

**Introduction:**

Sensory processing challenges are commonly encountered in pediatric patients, particularly in those who are neurodivergent. We previously developed a novel clinical pathway (named “Sensory Pathway”) which aimed at improving patient care for those with sensory barriers via staff training, provision of sensory toolkits and early integration of families throughout the hospital stay. We hypothesized that utilization of this pathway will result in improved patient experience and provide valuable feedback to improve care.

**Methods:**

A voluntary survey was made available to all patients who utilized this resource as part of our hospital wide patient satisfaction survey. Qualitative data was coded using open coding as part of the constant comparison method data using NVivo 12 for Windows software for analysis. Software was used to create word clouds and clusters for visualization, which confirmed the themes and patterns that were noted from initial open coding.

**Results:**

Between 2021 and 2022, surveys were obtained from 160 patients who utilized the Sensory Pathway. More than 50% reported that the most helpful components of the pathway were the approach by the staff and sensory tools. The three major themes identified from the survey were (1) Tools and techniques that benefited their children; (2) Positive interactions and communication with the hospital staff, and (3) Suggestions for future improvement.

**Conclusion:**

The survey results highlight the importance of having tools readily available to aid with sensory regulation and comfort of patients during healthcare encounters, the value of a positive patient and staff encounter, as well as opportunities for improvement.

## 1 Introduction

Sensory processing challenges are commonly encountered in pediatric patients, particularly in those who are *neurodivergent*. Neurodivergent is a common nonmedical umbrella term that is used to describe when someone's brain processes or learns differently (1) and can include diagnoses such as Autism Spectrum Disorder (ASD), Down Syndrome, Fetal Alcohol Syndrome and Attention-Deficit/Hyperactivity Disorder (ADHD) (2–4). Co-morbid conditions frequently accompany these diagnoses leading to the need for regular healthcare encounters (5). These patients often have unique sensory, social and communication profiles that can become a barrier to medical diagnosis and management. The sensory challenges can range from hypersensitivity to certain inputs (for instance, bright lights, loud noises and intense smell) or to having difficulties with filtering irrelevant external stimuli that becomes magnified when they are ill and in a different environment. We have previously demonstrated that staff in the healthcare environment feel unprepared and unequipped to accommodate the unique needs of these patients (6). Because of our primarily neurotypical healthcare culture, individuals with sensory processing challenges not only face barriers to appropriate medical care, they are also at higher risk for a negative care experience. In a recent study by Mazurek et al. (7), autistic adults reported lack of provider recognition of their sensory needs, lack of knowledge specific to their autism diagnosis, poor communication and lack of rapport. Similarly, in another study by Raymaker et al. (8), autistic adults and adults with and without other disabilities perceived greater barriers to medical care, particularly as it relates to their sensory differences and the patient-provider communication. Together, these studies highlight the need to rethink our healthcare delivery system to better meet the unique needs of our neurodivergent patients.

To address the barriers faced by patients with sensory challenges, we developed the Sensory Pathway at Children's of Alabama (9). Components of the Sensory Pathway consist of staff training, provision of sensory tools and social stories as well as adaptation to the patient intake and admission flow. Staff training was focused on identification of a patient with sensory processing difficulties, different methods of engagement and preventive strategies, effective communication strategies, use of toolkits and storyboards as well as de-escalation techniques during a sensory crisis. The training was provided by members of the sensory task force (pediatric intensivist, nurse educators and child life specialists) to all staff (physicians, nurses, respiratory therapist etc.) in the individual units (on average 4–6 sessions, 60-min sessions per unit). Sensory toolkits which included items such as noise canceling headphones, fidget tools, light spinners and weighted lap pads were provided and made easily available for each unit. Storyboards were written in precise and sequential way using simple and literal language for various procedures (for instance intravenous line placement) or encounters (for instance a trip to the radiology scanner) to help pre-condition a child to the event. If feasible, environmental adaption (placement in quieter room for noise sensitive child) or adjustment of procedure scheduling were made for patients placed on the Sensory Pathway.

We hypothesized that utilization of the pathway will result in improved patient experience and provide valuable feedback to improve care.

## 2 Materials and methods

### 2.1 Patient survey

A patient survey (see Appendix 1) was made available to all patients and their families who utilized this resource as part of our hospital wide patient satisfaction survey. These surveys were voluntary, and all comments received were reviewed by members of the sensory task force to allow for staff feedback and pathway modification if necessary.

### 2.2 Ethics

Patient satisfaction surveys were given routinely for any patient admitted to the hospital, and the Sensory Pathway survey was offered as part of this hospital wide survey. Because this project was part of a Quality Improvement (QI) initiative performed to improve patient experience for children with sensory barriers, informed consent was not obtained from the families. Guidelines for reporting QI initiatives published by the SQUIRE Development Group were consulted for this manuscript (10).

### 2.3 Qualitative data analysis

Qualitative data from the surveys were uploaded into NVivo 12 for Windows (Release 1) software for analysis. Two members of the research team independently coded the data using open coding as part of the constant comparison method (11) adding initial codes, then comparing and revising them as they discovered themes and patterns in the data. The researchers then met to gain consensus. After discussing various themes, they revisited the data, using the software to create word clouds and clusters for visualization. These methods further confirmed the themes and patterns that the researchers had noted from initial open coding.

## 3 Results

### 3.1 Quantitative results

Between 2021 and 2022, 160 patient surveys were obtained from patients who triggered the Sensory Pathway. Of the 160 visits, 151 patients had a previous visit to the same hospital in the past. Only 9 patients were new to the hospital system. For those who had been previous patients, we asked if the patient experience was improved, the same, or worse with the initiation of the Sensory Pathway. Sixty eight percent and 5% reported that their visit was improved and the same, respectively. One patient reported a worse experience, while 25% did not respond to this question. More than half of the patients reported that the most helpful components of the pathway were the approach by the staff and sensory tools (59% and 57%, respectively). Seventeen percent acknowledged the social story to be most helpful**.**

### 3.2 Qualitative results

**Figure 1:**
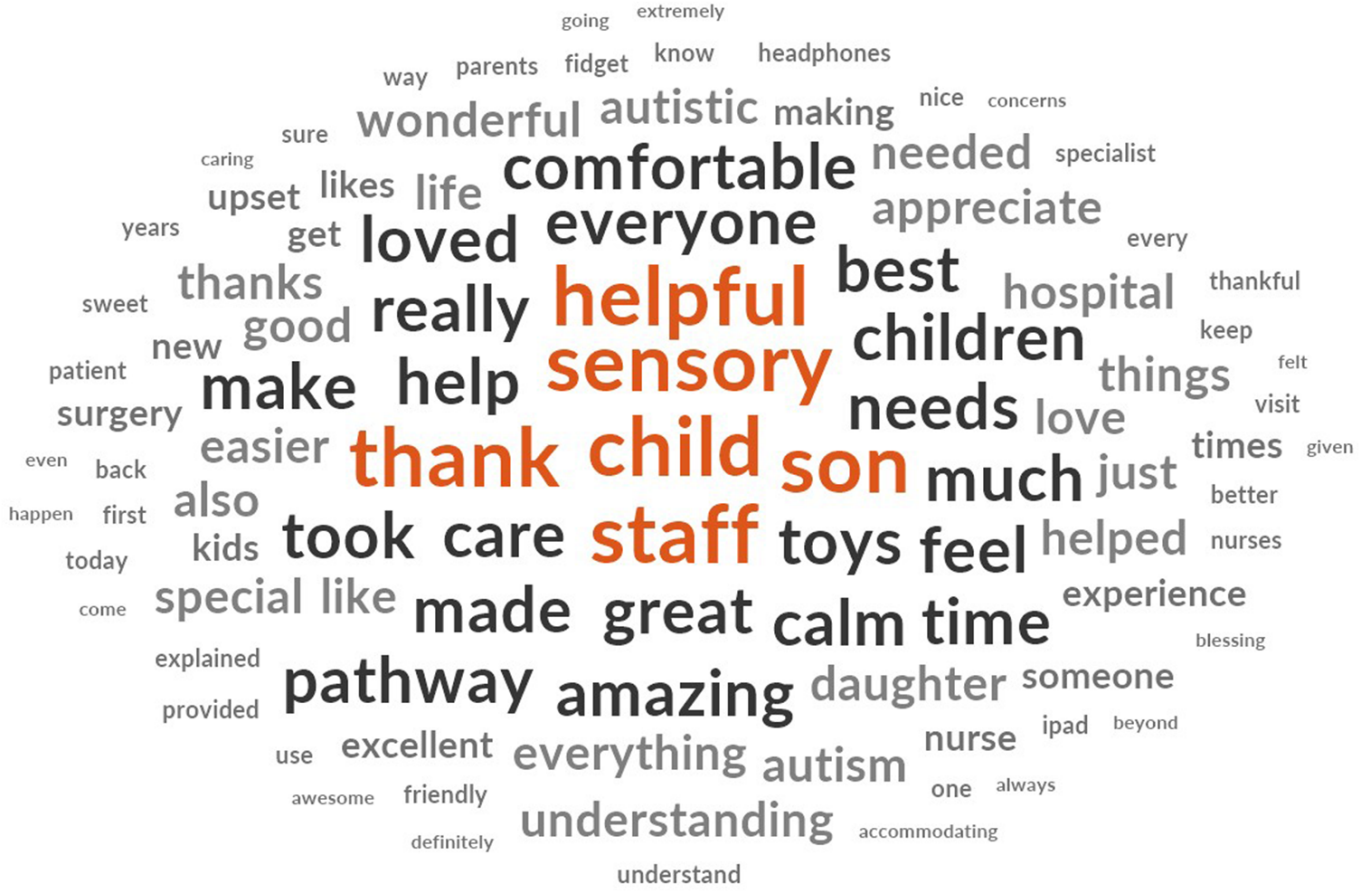
Most used words in the Sensory Pathway patient satisfaction survey.

Figure 1 is a word cloud generated from all the surveys that highlights the most used words.

The three major themes identified in the dataset were: (1) Tools and techniques that benefited their children, (2) Positive interactions and communication with the hospital staff, and (3) Suggestions for future improvements.

#### 3.2.1 Theme 1: sensory tools and techniques

When asked to provide comments on the questionnaire, many participants shared that a variety of tools and techniques were helpful in easing medical visits for their child. The following sub-themes were identified: (1) Sensory pathway in general; (2) Specific sensory toys or tools, and (3) Techniques used.

##### 3.2.1.1 Sensory pathway

In the comment section of the questionnaires, participants often reported that the sensory pathway was helpful during their child's medical visit. The use of the pathway was mentioned 25 times. When mentioned, participants described the sensory pathway as “help(ing) to keep him calm”, aiding to make “the visit (go) easier”, and “calm(ing) all of our child's fears”. Other words to describe the sensory pathway included superlatives/adjectives such as “loved” (*n* = 3), “great” (*n* = 3), “enjoyed”, “likes the items”, “helpful” (*n* = 6), “awesome”, “impressed” (*n* = 2), “useful” (*n* = 2), and “amazing idea”. None of the participants shared any negative comments about the sensory pathway.

##### 3.2.1.2 Specific sensory tools

The overwhelming majority of the referenced tools were simple toys; however, technology-enabled tools were also mentioned. Of the 35 specifically mentioned toys or tools, only several were mentioned more than once. These included noise cancelling headphones (*n* = 3), iPad or tablet (*n* = 6), fidget items (*n* = 4), weighted blanket/lap pad (*n* = 5), music (*n* = 2), bubbles (*n* = 2), and balls (*n* = 2). Tools and toys mentioned only once included Rubik's cube, puzzles, massager, locks, Lego® blocks, Halloween costume, crayons, clicking cube, and chewy toys. Two participants stated that they wished more sensory toys were available. Further, another participant recommended the addition of a sound machine.

##### 3.2.1.3 Techniques

The participants shared 19 techniques that were helpful during their child's visit. Several participants cited distraction (*n* = 3) as a helpful technique as noted by these comments: “staff (kept) him distracted”, “helped him count the butterflies”, and “talk(ed) about NBA”. Other cited techniques that participants found helped during their child's visit included listening (*n* = 2), explaining the reason for the visit (*n* = 3), making the feel patient comfortable, building rapport (*n* = 3), use of a separate waiting room, speaking directly to the patient, reassuring the patient, providing playtime, using a pen as a “magic wand”, and the use of a click and breathe technique.

#### 3.2.2 Theme 2: staff interactions

Many participants expressed that they had spent extensive time at the hospital to address healthcare issues and that dealing with their child's medical diagnosis and sensory processing barriers was especially challenging in this setting. They cited various ways in which the hospital staff made their visit smoother, including the following sub-themes: (1) Superlatives and adjectives to describe interactions with staff; (2) Descriptions of helpfulness and (3) Descriptions of kindness and caring toward their child or their family.

##### 3.2.2.1 Superlatives/adjectives

Coding revealed that participants used various superlatives or adjectives of praise over 100 times to express their satisfaction with the visit. These included words such as “amazing,” “excellent,” “awesome,” and “great,” many of which were capitalized and/or followed by numerous exclamation points. Superlatives included statements such as “This hospital and Emergency Department is the best for our family,”; “We have received the best NICU care” and “Dr. X is the best ENT in the state of Alabama. I would probably say the whole U.S. He's the best ENT there is.” There were also 33 instances of participants using the words “thank you” or “thanks” to express appreciation.

##### 3.2.2.2 Helpfulness

Families reported that that providers were helpful in numerous ways. Many expressed that hospital staff found ways to calm their child and to allay their anxiety. For example, one participant claimed, “Our CA was very friendly and told my child everything that was happening in a calm manner, so he didn't get upset.” Some participants noted that staff eased their anxiety as well, such as one who explained, “Our nurse that took him back to the OR was so sweet and helped him count the butterflies on the way so that he didn't get upset. And she even stopped by afterwards to calm us as parents to let us know he did great!”. One participant stated that all staff employed the calming strategy: “Each talked calmly to our child to assist with reducing her stress.” This emphasis on calming was clearly important to participants, as summed up by one: “I would recommend [hospital] to anyone that doesn't have a special needs child and especially if you do have a special needs child. They listen to you and try their very best to make it a calm experience.”

Another helpful trait noted by participants was the staff's effort to be informative about what was taking place during the child's appointment. One reported that it was this effort to provide clear information that created the calming effects: “Everyone communicated to us every step of the way and provided updates to help calm us as parents.” Another participant described how a provider helped during discharge: “She explained things so that we could retain them and wrote details for us in the paperwork (timing of medicine) to help.” Participants especially appreciated how staff communicated with the child. One stated, “My son has Down syndrome and the staff took excellent care of him, spoke directly to him, explained what they were doing.” Another cited the same kind of notable interaction: “Our son is 13 and primarily nonverbal. Your staff (audiologist, nurses, NP, and physician) spoke to him and explained everything that was happening and what they were doing.” Another participant praised a provider specifically for communication skills: “Just would like you to know the doctor explained everything to me, and to my child who is also on the autism spectrum in a way that we both understood and made it very clear.”

Participants also praised providers for their listening skills, using phrases such as “took time to listen” and “very receptive to listening to us” to express satisfaction. One participant explained: “Our son is very difficult at times to choose the proper sensory procedures or supplies but the staff seem more open to listening to our concerns for his safety. We appreciate the staff listening to the difficulties facing our child.” Another was grateful that “we were in and out very quickly with the doctor listening to our concerns and helping us to understand what would make us need to come back. And we just overall felt like we were taking very good care of.” In several instances, even if some parents did not feel like they had enough time with the doctor, they were still satisfied overall, as one noted: “We really appreciated the attention of the staff, and…we wish we had more time, but I understand they see a lot of kids, but we did feel heard.” Overall, participants stated that clear communication of important information, combined with the willingness to listen carefully to them and/or their children, contributed to a calm feeling that was tremendously helpful for all.

##### 3.2.2.3 Kindness and caring

Many participants noted an overall attitude of kindness and caring from staff during their visit. Some of these comments were direct references, such as “The nurses were very caring and sweet to my child,” and “Every member of the hospital staff was kind.” Others used different adjectives to illustrate this attitude, with words such as “friendly” (*n* = 8), “sweet” (*n* = 8), and “patient” (*n* = 5) to describe the care they received. One parent described actions taken by staff that showed they cared: “She was the best nurse we have ever encountered because she was observant and proactive. She thought of our son's needs and provided books, toys, supplies, etc. without hesitation even before we thought of what we needed. It was so nice to have her attentiveness to detail because we got admitted somewhat unexpectedly after a CT scan and didn't have a lot of basic supplies with us.” It was also important to families that staff recognized their children's disabilities, with one stating that they appreciated that the staff “understand that she is special.” Another participant explained: “Everyone was very sweet and considerate of our and her disability. They got down on her level and spoke to her and explained everything to her. They also tried to find female doctors and nurses to help because she is more comfortable with them instead of males.” Finally, one parent summed up the overall effect of the attitudes of staff toward patients with this statement: “We appreciate that at [hospital], being ‘different’ isn't that different at all.”

#### 3.2.3 Theme 3: future improvements

Parents who made suggestions for improvement primarily focused on the duration of wait times. For example, one parent acknowledged that their only complaint was the emergency room wait time, while another claimed: “The wait time was too long for a child with autism and ADHD in crisis mode.” One parent suggested: “I think kids with autism that don't understand they have to wait to eat need to be higher up on the time priority, rather than later in the day because they don't understand that they can't eat for so long and they don't understand why they're having to wait for so long.” In a similar incident, a parent offered a perspective about *why* they had to wait: “The only complaint we had is we came in for a short procedure. We were there a little bit before 8:30 but they almost didn't take her back till noon, and we realize that they kind of have an order that they go by. A lot of times, they take the younger children. My daughter is a little bit older; she is 6. However, she does have special needs.”

Some suggestions for improvement were based on favorable overall comments. For instance, parents who found the sensory tools helpful suggested that the diversity of tools should be increased, and that they might be offered to children earlier in the visit, for instance during triage. Three parents named specific staff members who had been rude, but they acknowledged that the rest of the staff had exemplary communication. Two claimed that the bright lights in certain rooms made their children uncomfortable, but they also recognized that the lights were needed for proper care. Only two participants claimed that unfavorable experiences made them decide not to return to the facility for future care.

## 4 Discussion

For many neurodivergent patients and their families, poor recognition of the patients' unique sensory needs and ineffective communication often results in unfavorable hospital experiences (12). The Sensory Pathway aims to create an environment and healthcare experience that promotes inclusivity and removes barriers that stand between proper assessment, diagnosis, treatment, and overall positive care experiences for patients with sensory processing differences (9). As reflected by the 94% of the patients in this study cohort who had a previous hospital encounter, many patients with sensory processing differences return to the healthcare setting due to co-morbid conditions. The Sensory Pathway program aims to provide positive care experiences that will lay the foundation for future healthcare experiences.

Patients and families who are placed on the Sensory Pathway are provided access to staff who have been trained to recognize sensory processing challenges and utilize adaptive care approaches to combat potential sensory overload during healthcare experiences. During visits, caregivers are asked questions about their child's sensory sensitivities and needs. Sensory specific resources are also made available to promote sensory regulation and positive coping.

In this survey, more than half of the patients reported that the most helpful component of the pathway was the availability of sensory tools. Sensory tools made available through the Sensory Pathway are selected based on their potential to assist with sensory regulation of the various internal and external senses. Tools may offer a specific sensory input for individuals with hypo-sensitivities or sensory seeking needs or guard against sensory input for hyper-sensitivity or sensory avoidance (13). They can also be leveraged as both comfort and distraction tools (14). Since the sensory needs of individuals can vary greatly, we found it beneficial to have a variety of tools available for patients and families to select from. We provide headphones, fidget tools, weighted lap pads, mobile sensory stations and other sensory tools which were paired to the patients based on their sensory needs and parental input. This was supported by our qualitative analysis in which families cited the positive impact of a wide variety of sensory tools with no clear preference for any one tool. Often families use similar items at home but because of the urgent nature of the hospital visits, these tools are not always brought with them to the hospital.

However, in certain instances, a patient might need very specific tools unique only to them. This is especially true for autistic patients who might have restricted interest (15). For instance, a patient was noted to be very overwhelmed at the hospital, and further questioning revealed that he typically wore a Hulk Halloween costume at home, which was his primary comfort item. The team found a matching costume for the patient to wear during his visit, and this costume served as a point of familiarity for the patient. This incident reinforces the importance of maintaining baseline routines and incorporating the patient's interests into their healthcare experiences (16).

In addition to physical resources such as sensory tools and toys, it is critical to utilize techniques such as diversion or distraction to engage the patient. These techniques have been found to be widely effective in reducing reported and observed pain as well as promoting positive coping and compliance during medical events (17–19).

One of the major themes identified in this study relates to the importance of a positive patient-staff interaction. Many caregivers of autistic children report stress and concern around the unempathetic behaviors and reactions of others (20). In the Sensory Pathway, we focused heavily on staff education, in how to recognize a sensory patient, best method of engagement, use of preventive measures to avoid sensory overload, and non-pharmacologic de-escalation techniques.

A positive interaction often hinges on effective communication. Thus, a primary emphasis in our training is on communication between staff, staff to patient, and staff to caregivers. Lack of communication in the healthcare setting often results in failure to gain a comprehensive understanding of the patient and family as a whole and their specific needs. For caregivers of patients, this communication gap can introduce feelings of emotional distress that present as anger, frustration, anxiety, and sadness (21). Ineffective communication can also influence the patient's ability to appropriately process their healthcare experience (22). For staff-to-staff communication, in addition to verbal communication between the healthcare team regarding the patient's sensory profile, we also utilize visual notifiers and reminders within the environment and in the patient's electronic medical record. Information gathered from caregivers about the patient's sensory specific needs is communicated among the healthcare team throughout the patient's care experience and especially during transitions of care such as at shift change handoff.

Heighted anxiety is common among individuals with autism and ADHD and is widely seen in individuals visiting the healthcare setting (23). Our staff to patient communication involves adjusting communication strategies when working with patients to help reduce anxiety by ensuring they can appropriately process information and commands being given. During stressful situations, emotional distress may be exacerbated if an individual perceives that they do not possess the appropriate amount of information to understand what is happening or will happen in that moment. Providing relevant information offers a sense of control and predictability which in turn can aid in reducing anxiety and increasing positive coping and compliance (24). Staff are encouraged to use communication strategies such as keeping language simple, specific, and concrete. Visual resources including social stories are also available to help patients visualize what they may experience during specific procedures or healthcare encounters. We found that that story boards can be effectively utilized to pre-condition patients to certain procedures such as intravenous line placement, urinary catheter placement, laceration repair, radiographic imaging etc. This is supported by others who have shown that story boards can be used successfully to alleviate anxiety (25).

Lastly, in our staff-to- caregiver communication, we emphasize that it is important to not only ask questions surrounding the medical complaint but ask specific questions pertaining to the patient's baseline behaviors, sensory sensitivities, preferences, and more. By proactively gathering this information from caregivers, staff are better equipped to make modifications to the environment and provide sensory specific resources that can aid in reducing the potential for sensory overload during the patient's healthcare experience.

Evaluation of feedback from our stakeholders is a valued component of continuous program development to ensure efforts to improve care are being carried over to the patient and family experience. As with any program, when reviewing feedback, opportunities for optimization were identified. Suggestions centered largely around shorter wait times for patients, particularly those requiring nil per os (NPO, nothing by mouth) status for surgeries or sedated procedures. Opportunities to incorporate special needs such as sensory vulnerabilities into scheduling algorithms and processes may assist in reducing anxiety and agitation brought on by extended wait times. In addition, the expansion of sensory rooms and sensory safe environments may help in guarding against sensory overload as patients and families wait (26, 27).

Neurodivergent patients have unique barriers when they are within the hospital setting. These social, communication and sensory barriers lead to not only poor patient experience, but potentially to misdiagnosis and mismanagement. While this program was implemented in a Children's Hospital, given the wide spectrum of neurodivergent patients that spans the entire age range, this pathway is likely to benefit adult patients as well. Patients with traumatic brain injury, post-traumatic stress disorder (PTSD), dementia or the elderly often have sensory barriers that are further amplified when they are ill (28–30). The same considerations in how we meet engage with our neurodivergent patients should be given in all medical settings, whether in-patient, in the emergency room or in a community clinic. The goal of the Sensory Pathway is to mitigate these challenges and to improve the overall healthcare experience of neurodivergent patients. The survey results highlight the importance of having tools readily available to aid with sensory regulation and comfort of patients during healthcare encounters, the value of a positive patient and staff encounter, as well as opportunities for improvement. As frequent healthcare encounters are a reality for many neurodivergent individuals with sensory processing differences, increasing staff's understanding of sensory inclusive care through educational opportunities must be prioritized to set a continued positive shift in mindset and approach to care with this population. Successful implementation of this pathway required buy-in from all the major stakeholders, starting from the hospital leadership to unit directors, and to frontline staff. It was not limited to a particular group of providers (for instances only nurses or child life specialist) as it was crucial for anyone who might have an encounter with the patient from the moment, they entered the hospital to be trained. Frequent check-in with sites to ensure that staff training was up-to-date, tools were available, and no changes in patient flow was needed was also key after the initial implementation.

In conclusion, it is critical that we continue to create sensory diverse and inclusive environments where patients, families, and staff alike can be successful and feel empowered to master their healthcare experience.

## Data Availability

The original contributions presented in the study are included in the article/**Supplementary Material**, further inquiries can be directed to the corresponding author.
