# Supplementary material for: Neurodivergent patient experience in a tertiary children's hospital-a qualitative analysis
Source: Front Pediatr. 2024 Jul 16;12:1427433. doi: 10.3389/fped.2024.1427433 (PMC11286462; doi:10.3389/fped.2024.1427433)
Supplement: Supplementary file 1 [file Datasheet1.pdf]

Sensory Pathway Survey

1. If your child has been admitted to the hospital in the past, do you feel that the patient had improved care and treatment due to the use of the sensory alert pathway during this visit?

    1. Yes
    2. No
    3. The same
    4. Don't know

2. What part of the pathway do you believe was most helpful for your child?

    A. Hospital Staff

    B. Social Stories for procedures

    C. Supplies/tools

3. General Comments:

____

____

____

____

____

____

____

____

____
